# Dual functional oyster shell-derived Ag/ZnO/CaCO_3_ nanocomposites with enhanced catalytic and antibacterial activities for water purification†

**DOI:** 10.1039/c9ra08960h

**Published:** 2019-12-13

**Authors:** Lin Chang, Yan Feng, Bingqing Wang, Xiaoyu Huang, Da-Peng Yang, Youguang Lu

**Affiliations:** Department of Preventive Dentistry, School and Hospital of Stomatology, Fujian Medical University Fuzhou 350002 Fujian Province PR China fjlyg63@163.com +86-591-83720599 +86-591-83736429; Key Laboratory of Stomatology & Fujian Provincial Engineering Research Center of Oral Biomaterial, School and Hospital of Stomatology, Fujian Medical University Fuzhou 350004 Fujian Province PR China; College of Chemical Engineering and Materials Science, Quanzhou Normal University Quanzhou 362000 Fujian Province PR China yangdp@qztc.edu.cn +86-595-22199816 +86-595-22199816

## Abstract

Transforming biological waste into high value-added materials has attracted extensive current research interest in the wastewater treatment field. In this study, oyster shell-derived CaCO_3_ was used as a solid support to construct efficient Ag/ZnO/CaCO_3_*via Cacumen platycladi* extract. As compared to binary Ag/CaCO_3_ and ZnO/CaCO_3_, the as-obtained Ag/ZnO/CaCO_3_ exhibits excellent performance for the reduction of 4-nitrophenol (4-NP) with a reduction rate of 97.6% within 8 minutes. Furthermore, Ag/ZnO/CaCO_3_ exhibited superior antibacterial activity in inhibiting the growth of *Escherichia coli* (*E. coli*) and *Staphylococcus aureus* (*S. aureus*) with minimum inhibition concentrations (MICs) of 60 μg mL^−1^ and 30 μg mL^−1^, respectively. The observation of the superior activity for the reduction of 4-NP and antibacterial test of Ag/ZnO/CaCO_3_ can be attributed to the synergistic effect of Ag, ZnO and the oyster shell-derived CaCO_3_. This study provides a strategy in developing highly efficient catalysts for the reduction of 4-NP and antibacterial agents based on the oyster shell-derived CaCO_3_.

## Abstract 2

Oyster shell was used as an efficient template for synthesis Ag/ZnO/CaCO_3_ nanocomposites, which showed the enhanced catalytic and antibacterial activities.

## Graphical abstract

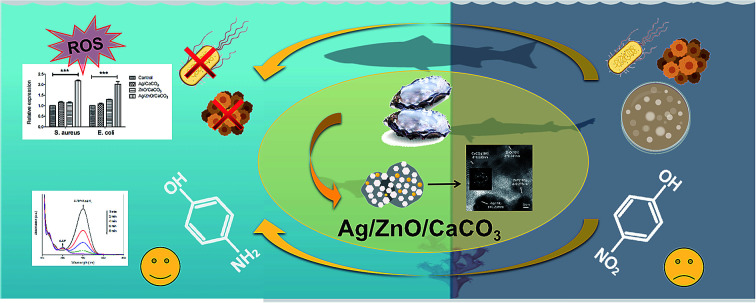

## 1. Introduction

The transformation of biological waste into high value-added materials has attracted extensive current research interest. Oyster shells, abundant but discarded in the coastal areas, have already been demonstrated to be attractive in some important fields such as wastewater treatment and regenerative medicine, due to their natural porous structure, abundance as well as rich biogenic calcium carbonate (CaCO_3_). Furthermore, the geochemical properties of biogenic CaCO_3_, such as its exchange capacity, high absorbability and reactive surface area make it ideal as a solid support to construct efficient bio-based materials for wastewater treatment.^1,2^ Although some examples have been reported in this area, the modification of organic compounds, including sodium dodecyl sulfate (SDS) and cetyltrimethyl ammonium bromide (CTAB), would tend to cause secondary pollution to bodies of water.^3^ Therefore, more explorations of oyster shell-derived materials for sustainable and green chemistry are still required.^4–6^

Recently, wastewater treatment based on semiconductor (photo) catalysis is regarded as a powerful method, as it can degrade the majority of harmful pollutants (such as 4-NP reduction) from wastewater. Until now, a variety of semiconductors, including ZnO, CuO as well as NiO, have been developed for the treatment of a wide range of organic/inorganic contaminants and pathogenic microorganisms from wastewater.^7–12^ Among these materials, ZnO is considered one of the most promising emerging semiconductors, and features advantages in terms of low cost, broad-spectrum, and environmental friendliness.^13,14^ In particular, the suitable redox potential of ZnO makes it degrade the majority of harmful pollutants from wastewater. However, the catalytic activity for wastewater treatment over bare ZnO still suffers from limited reusability and ease of aggregation.^15^

It is well known that noble metals play an important role in semiconductor-based catalytic activity since they can provide active redox sites. Based on previous studies, a noble metal (*e.g.*, Pd, Pt, Au, Rh, and Ag) is usually used to promote the ZnO catalytic performance.^16–19^ As a typical noble metal, Ag has successfully attracted extensive current research interest in the wastewater treatment field, since Ag provides sufficient active sites for the degradation of harmful pollutants and accelerates the degradation of the pollutant.^20–23^ By far, various kinds of synthetic strategies, such as electrochemical, decomposition, microwave assisted technology and wet chemical, have been designed for the fabrication of binary Ag/ZnO, but they are generally costly, flammable and toxic.^24–30^ Therefore, developing an efficient and environmentally friendly route to prepare binary Ag/ZnO is urgently required. Recently, the plant exact is considered to be one of the most efficient methods, since the unique biological molecules (such as sugars, polyphenols and flavonoids, plant extracts, especially *Cacumen platycladi* extracts) can act as reducing, covering and stable agents in the synthesis process.^31–34^

In this work, Ag/ZnO obtained from *Cacumen platycladi* extract was coated onto oyster shell-derived CaCO_3_, and used for the reduction of 4-NP and as an antibacterial agent. It was found that the as-obtained Ag/ZnO/CaCO_3_ exhibited excellent performance for the reduction of 4-NP and as an antibacterial agent, as compared to that of binary Ag/CaCO_3_ and ZnO/CaCO_3_. The observation of the activity for the reduction of 4-NP and the antibacterial test of Ag/ZnO/CaCO_3_ can be ascribed to the synergistic effect of Ag, ZnO and the oyster shell-derived CaCO_3_.

## 2. Materials and reagents

### 2.1 Materials

The oyster shells and *Cacumen platycladi* used in this work were acquired from Quanzhou Normal University, China. Zinc nitrate hexahydrate (Zn(NO_3_)_2_·6H_2_O) and silver nitrate (AgNO_3_) were purchased from Sinopharm Chemical Reagent Co., Ltd. (China). 4-NP (C_6_H_5_NO_3_) was produced from Qigang Rd., Nanqiao Town, Fengxian, Shanghai. Sodium borohydride (NaBH_4_) was obtained from Xilong Scientific Co., Ltd. Glutaraldehyde was purchased from Tianjin Ruijinte Chemical Co., Ltd. Nutrient agar and nutrient broth were acquired from Shanghai Bowei Biotechnology Co., Ltd. Bacteria were obtained by the Second Affiliated Hospital of Fujian Medical University (Quanzhou). The reactive oxygen species assay kit was purchased from Biyuntian Biotechnology Co., Ltd. All chemicals were of analytical grade and the water used in this work was deionized (DI) water.

### 2.2 Synthesis of Ag/ZnO/CaCO_3_ nanocomposites

First, the oyster shells were rinsed thoroughly with tap water until all dirty matter was removed. The washed oyster shells were again rinsed twice with DI water, and were dried at room temperature for 24 hours. The dried oyster shells were powdered with a grinder, and sieved through a 200 mesh sieve, and then stored in a desiccator for further research. The material thus obtained was the oyster shell-derived CaCO_3_.

2 g of CaCO_3_ was dispersed in 50 mL of 0.5 mol L^−1^ Zn(NO_3_)_2_·6H_2_O solution under magnetic stirring for 24 hours. The reaction was vacuumed to take the solid powder, dried at room temperature, and then collected in a crucible. The ZnO/CaCO_3_ nanocomposites were obtained after calcination at 600 °C in a muffle furnace for 4 hours under a heating rate of 5 °C min^−1^. Thereafter, 1 g of the ZnO/CaCO_3_ nanocomposite was immersed in 10 mL of 0.1 mol L^−1^ AgNO_3_, and 10 mL of *Cacumen platycladi* extract was added. The mixture was combined with magnetic stirring for 48 hours without light, and then thoroughly washed with ethanol and water, and dried. For comparison, Ag/CaCO_3_ nanocomposites were prepared under similar experimental conditions without the Zn(NO_3_)_2_·6H_2_O solution.

### 2.3 Material characterization

X-ray diffraction (XRD) measurement of the samples was conducted with a Bruker D8 Advanced System over a 2*θ* range from 10° to 80° with a Cu Kα radiation source produced at 40 kV, 40 mA, with a scanning speed of 10° min^−1^. The morphological structures of the samples were examined by a field-emission scanning electron microscope (FESEM, Zeiss, Germany), which was combined with energy dispersive spectroscopy detectors (EDS, Zeiss, Germany) at an accelerating voltage of 1.5 kV. TEM and HRTEM images were taken on a Tecnai F30 FEI transmission electron microscope under a 300 kV operating voltage. X-ray photoelectron spectroscopy (XPS, Thermo Fisher Scientific ESCALAB 250Xi, USA) of the samples was performed with an Al Kα X-ray source. Thermogravimetric analysis (TGA, TA Instruments Q50, US) of the samples was conducted at a heating rate of 5 °C min^−1^ in air.

### 2.4 Catalytic reduction of 4-NP

To investigate the catalytic reduction performance of the sample to 4-NP, 4 mL of freshly prepared NaBH_4_ solution (0.33 mM) and 2 mL of 4-NP solution (1 mM) were mixed in 25 mL of DI water at room temperature. Then, 2 mg of the Ag/ZnO/CaCO_3_ nanocomposite was added to the mixture. A portion of the mixture was taken at intervals of 2 minutes before and after the addition of the nanocomposite, and the concentration of 4-NP was monitored using a UV-Vis spectrophotometer (METASH UV-9000S) to measure the absorbance at 400 nm. Ag/CaCO_3_ and ZnO/CaCO_3_ were each added as a control under the same experimental conditions. In addition, a recycling experiment was performed to evaluate the reusability of the Ag/ZnO/CaCO_3_ nanocomposite. The sample was centrifuged before each cycle test, washed several times with DI water and dried, and the test was repeated five times.

### 2.5 Antibacterial test

*E. coli* (ATCC 25922) and *S. aureus* (ATCC 25923) obtained from Research Laboratory of the Second Affiliated Hospital of Fujian Medical University were utilized to evaluate the antibacterial potential of the samples. *E. coli* and *S. aureus* were cultured in Luria–Bertani (LB) nutrient agar media. After subsequent incubation at 37 °C for 24 hours, a series of experiments were performed to estimate the bacterial viability and antimicrobial potential.

#### 2.5.1 Agar plate experiment

The medium was rinsed several times with sterile water, and then the bacterial culture was suspended in a phosphate-buffered saline (PBS) solution at a final concentration of 10^8^ CFU mL^−1^. 50 μL of the suspension was applied to the solid LB medium plate. Moreover, tablets made of 70 mg of Ag/CaCO_3_, ZnO/CaCO_3_ or Ag/ZnO/CaCO_3_ nanocomposite were each placed on LB media and cultured in a 37 °C incubator for 24 hours. The antimicrobial and bactericidal properties of the samples were determined by observing the growth of the bacteria and measuring the size of the inhibition zone.

#### 2.5.2 Minimum inhibitory concentration (MIC) test

The bacteria were cultured to a logarithmic growth phase in the LB media, and then suspended in PBS at a final concentration of 10^8^ CFU. 1 mL of the bacterial suspension was added to 1 mL of PBS with different amounts of the Ag/ZnO/CaCO_3_ composite material to obtain bacterial solutions of different sample concentrations of 15, 30, 45, 60, 75, and 90 μg mL^−1^. The control groups did not have composite materials. The bacterial cultures were cultivated at 220 rpm at 37 °C for 12 hours. The growth of the bacteria was initially deduced by turbidity visualization. After that, the antibacterial activity of the Ag/ZnO/CaCO_3_ nanocomposite was supported with optical density analysis using a UV-Visible double beam spectrophotometer (Tecan Infinite 200 Pro, Austria) at a wavelength of 600 nm.

#### 2.5.3 Minimum bactericidal concentration (MBC) test

According to the results of the MIC experiment, different sample concentrations of 15, 30, 60, 120, 240, 480, and 960 μg mL^−1^ were prepared under similar experimental conditions, and two groups without materials were used as the control groups. The mixture was then set in a shaker incubator to promote the culture at 220 rpm for 12 hours. Moreover, 100 mL solutions were each transferred to the solid LB media, sprayed evenly, and then cultured at 37 °C for 24 hours to observe the colony growth on the plate.

#### 2.5.4 Characterizing the microstructure of bacteria

The bacterial cell death was analyzed by optical microscope and further by SEM. 2 mg of the sample was added to 10 mL DI water and ultrasonicated for 15 minutes. Afterward, 1 mL of the sample solution was added to 0.5 mL of the bacterial solution under shaking for 8 hours. The bacterial suspension was rinsed three times with PBS, fixed with 35% glutaraldehyde for 30 minutes, and dehydrated in a graded ethanol series (30, 50, 70, 90, 100%) for 15 minutes. Finally, bacterial droplets were dried on silicon wafers overnight prior to SEM observation.

#### 2.5.5 Reactive oxygen species (ROS) assay

To determine the production of ROS in cells, a 20 μM ROS assay kit containing DCFH-DA was used as a fluorescent probe. As a non-fluorescent dye, DCFH-DA is able to penetrate into the cells where it would be hydrolyzed to DCFH. In the presence of intracellular ROS, DCFH is converted into green fluorescent DCF for further analysis. In addition, *E. coli* and *S. aureus* were cultivated in LB medium with different materials of Ag/CaCO_3_, ZnO/CaCO_3_ and Ag/ZnO/CaCO_3_ for 8 hours. Thereafter, DCFH-DA was added and incubated for 6 hours at 37 °C. Finally, a Tecan Infinite 200 Pro fluorescence spectrophotometer was used for analysis.

## 3. Results and discussion

### 3.1 XRD of Ag/ZnO/CaCO_3_ nanocomposites

**Fig. 1:**
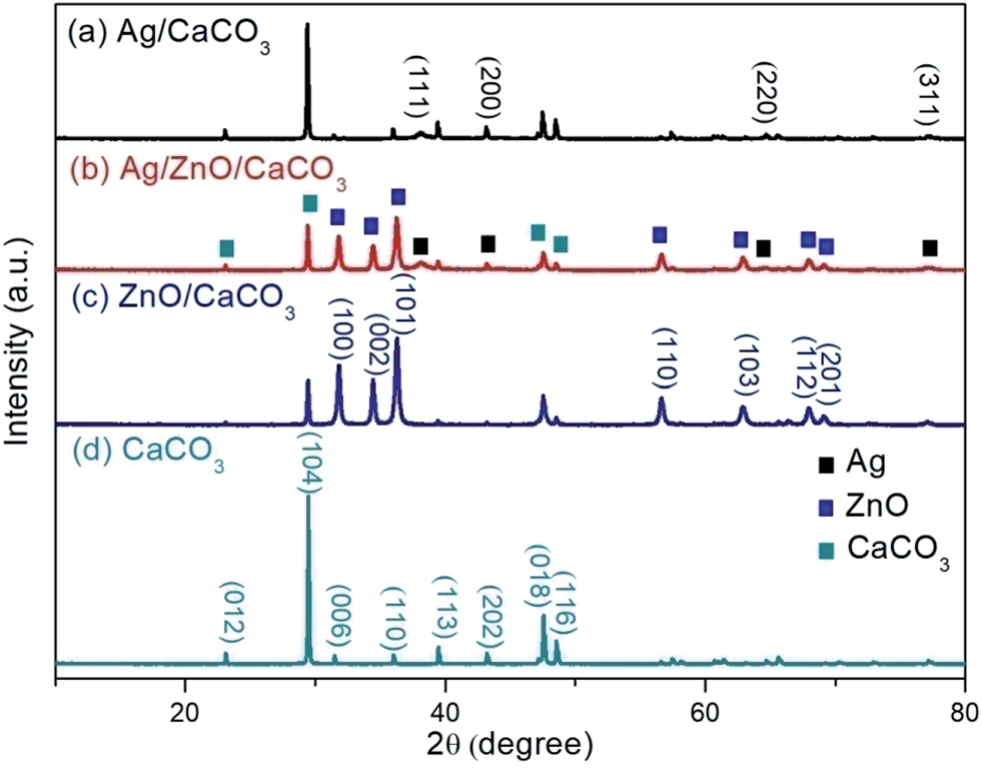
X-ray diffraction patterns of (a) Ag/CaCO_3_, (b) Ag/ZnO/CaCO_3_, (c) ZnO/CaCO_3_ and (d) CaCO_3_.

Ag/ZnO obtained from *Cacumen platycladi* extract was coated onto the oyster shell-derived CaCO_3_ (Ag/ZnO/CaCO_3_). For the XRD patterns of the as-obtained Ag/ZnO/CaCO_3_, the diffraction peaks at *ca.* 2*θ* degrees of 31.8°, 34.4°, 36.3°, 56.6°, 62.9°, 68.0°, and 69.1° correspond to the (100), (002), (101), (110), (103), (112) and (201) planes of hexagonal ZnO (JCPDS PDF # 36-1451). The diffraction peaks at *ca.* 2*θ* degrees of 38.1°, 44.3°, 64.4°, and 77.5° correspond to the (111), (200), (220), and (311) planes, which could be indexed to the face-centered cubic Ag (Fig. 1b). In addition, the diffraction peaks of oyster shell derived from CaCO_3_ were also observed (Fig. 1d), and are consistent with that of the bare calcite crystal CaCO_3_ (JCPDS PDF#05-0586). These results suggest that the as-obtained composites consist of the calcite crystal CaCO_3_, face-centered cubic Ag, as well as hexagonal ZnO.

### 3.2 SEM and TEM analysis

**Fig. 2:**
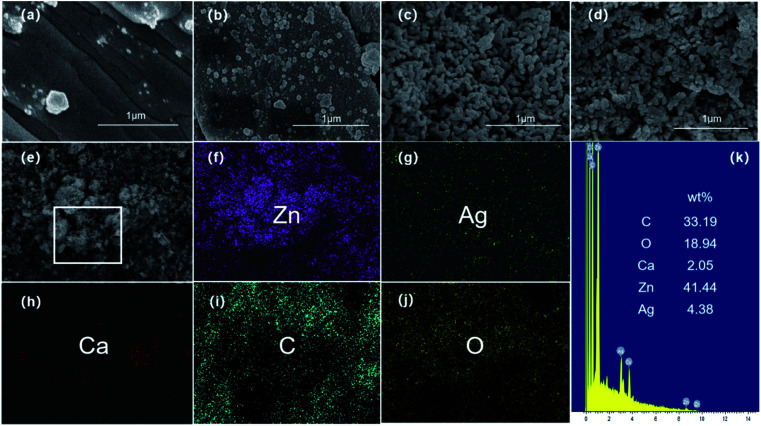
FE-SEM images of (a) CaCO_3_, (b) Ag/CaCO_3_ nanocomposites, (c) ZnO/CaCO_3_ nanocomposites, and (d) Ag/ZnO/CaCO_3_ nanocomposites. (e) FE-SEM image of the Ag/ZnO/CaCO_3_ nanocomposites and (f–j) corresponding elemental mapping images showing Zn, Ag, Ca, C and O in the selected area, (k) the EDX spectrum of the Ag/ZnO/CaCO_3_ nanocomposites.

The morphologies of Ag/ZnO/CaCO_3_ were observed by SEM, as shown in Fig. 2a–d. As compared to the bare oyster shell-derived CaCO_3_ with its smooth lamellar structure, there are a lot of granules with an average size at 100 nm, which is similar to that observed in the SEM image of Ag/CaCO_3_, distributed throughout the surface of Ag/ZnO/CaCO_3_. In corroboration, the EDX elemental mapping analysis of Ag/ZnO/CaCO_3_ confirms the existence and uniform distribution of the Ag, Zn, Ca, C, and O elements (Fig. 2e–k).

**Fig. 3:**
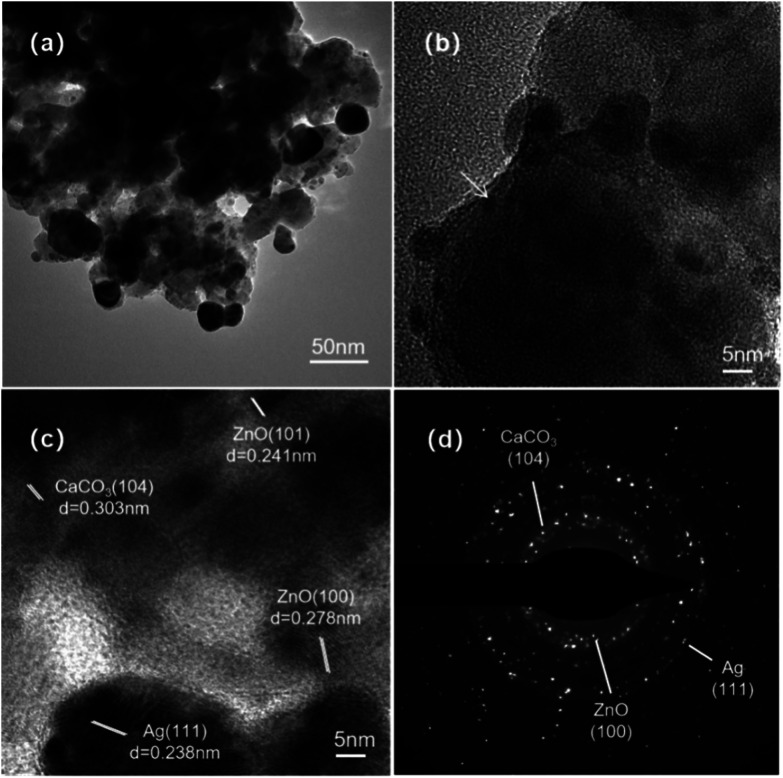
(a) Low magnification, (b) high magnification, as well as (c) HRTEM and (d) SAED images of the Ag/ZnO/CaCO_3_ nanocomposites.

The microstructure of Ag/ZnO/CaCO_3_ was further observed by TEM. As the TEM images of Ag/ZnO/CaCO_3_ show, two types of nanoparticles with different sizes at *ca.* 10 nm and 2 nm were evenly deposited on the surface of the CaCO_3_ sheet. In addition to the lattice fringes of 0.303 nm corresponding to the (104) crystallographic plane of the calcite crystal CaCO_3_, clear lattice fringes of 0.278 nm and 0.238 nm corresponding to the (100) plane of hexagonal ZnO and the (111) plane of face-centered cubic Ag, respectively, can be observed in the enlarged TEM image of Ag/ZnO/CaCO_3_, indicating that Ag/ZnO has been successfully coated on the surface of CaCO_3_ (Fig. 3).

### 3.3 XPS analysis

**Fig. 4:**
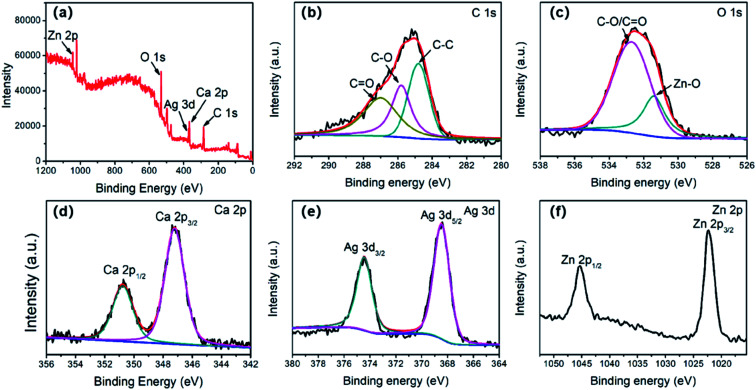
XPS spectra of the Ag/ZnO/CaCO_3_ nanocomposites: (a) XPS spectra of the survey scan, (b) C 1s, (c) O 1s, (d) Ca 2p, (e) Ag 3d, and (f) Zn 2p.

For further study of the surface information and electronic states of the Ag/ZnO/CaCO_3_, X-ray photoelectron spectroscopy (XPS) was carried out. The result of the wide spectrum indicated that the as-obtained Ag/ZnO/CaCO_3_ was composed of Ag, Zn, Ca, C, O, but there were no impurities (Fig. 4a). The XPS spectrum in the C 1s region of Ag/ZnO/CaCO_3_ shows three peaks at 284.8, 285.8 and 287.0 eV, ascribable to bonds of C–C, C–O, and C=O, respectively, in which the C–C was always clarified as a reference calibration (Fig. 4b). The XPS spectrum of Ag/ZnO/CaCO_3_ in the O 1s region can be deconvoluted into two peaks. The peak at 531.4 eV can be ascribed to O^2−^ in the Zn–O bonds, while the other peak at 532.6 eV can be attributed to the C–O/C=O species (Fig. 4c). There were two peaks with a peak spacing of 3.5 eV observed in the Ca 2p region of the XPS spectrum. The peak at 347.2 eV belonged to Ca 2p_3/2_, while the other peak at 350.6 eV reflected Ca 2p_1/2_ (Fig. 4d). The intensity ratio of the two peaks was nearly 2 : 1, which was in agreement with that previously reported in the literature.^20^ In addition, the peaks observed in Fig. 4e at the binding energies of 368.5 and 374.5 eV were attributed to Ag 3d_5/2_ and Ag 3d_3/2_, respectively. The splitting of the Ag 3d doublet was approximately 6 eV, which indicated the presence of metallic silver in Ag/ZnO/CaCO_3_, and is consistent with the results of XRD.^35^ Moreover, the characteristic peaks for Zn 2p_3/2_ and Zn 2p_1/2_ (at the binding energies of 1022.4 and 1045.4 eV, respectively) were found in the spectrum of Zn 2p with the splitting of 23 eV, and it can be inferred that Zn only existed in a divalent oxidation state in the sample.^36^

### 3.4 TGA analysis

**Fig. 5:**
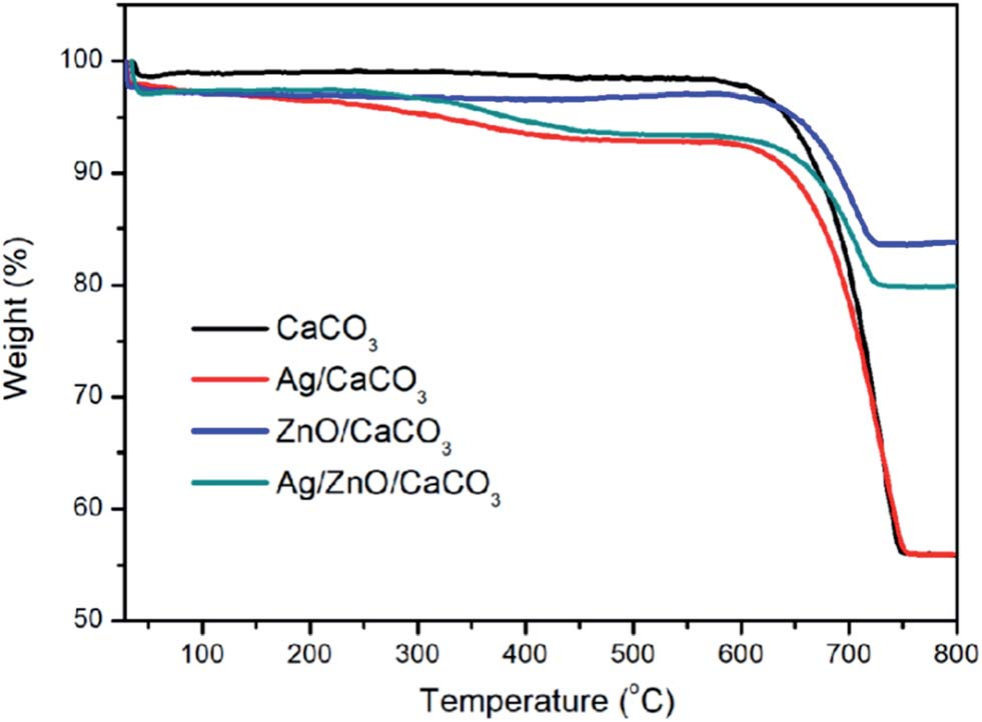
TGA patterns of CaCO_3_, Ag/CaCO_3_, ZnO/CaCO_3_ and Ag/ZnO/CaCO_3_.

The thermal decomposition of CaCO_3_, Ag/CaCO_3_, ZnO/CaCO_3_ and Ag/ZnO/CaCO_3_ were studied. As shown in Fig. 5, the loss of water from the samples occurred in the lower temperature range of 30–80 °C. It can be seen that a nearly 5% mass loss occurred from Ag/CaCO_3_ and Ag/ZnO/CaCO_3_ at around 350 °C, corresponding to the decomposition of organics in the arborvitae liquid. A dominant mass loss occurred at 630–730 °C due to the decomposition of CaCO_3_ and the loss of CO_2_. Once the temperature exceeded 750 °C, CaCO_3_ was completely converted into CaO and the decomposition tended to be stable. Since the oyster shell generally contained CaCO_3_ as the main calcium-based material, 600 °C was selected as a suitable calcination temperature for this study.

### 3.5 Catalysis reduction of 4-NP

**Fig. 6:**
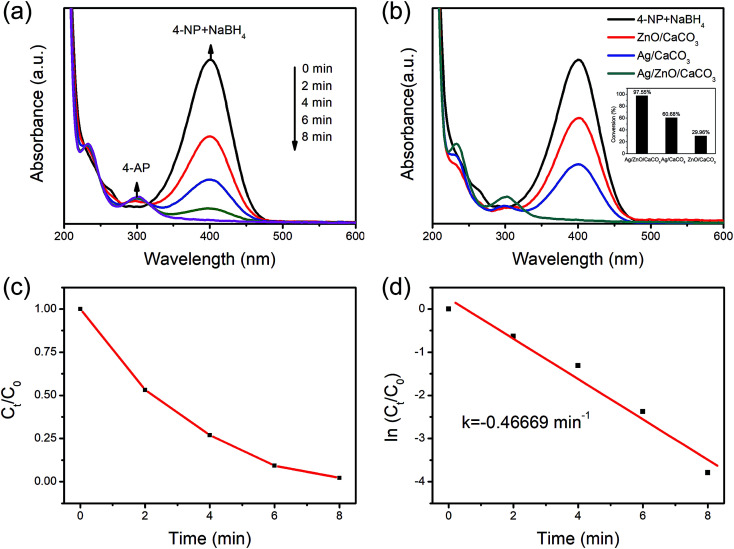
(a) Catalytic model of the Ag/ZnO/CaCO_3_ nanocomposites for 4-NP reduction, (b) different samples for 4-NP at 8 minutes, (c) and (d) plots of *C*/*C*_0_ and ln *C*/*C*_0_*versus* the reaction time, corresponding to the reduction of 4-NP catalyzed by the Ag/ZnO/CaCO_3_ nanocomposites.

**Fig. 7:**
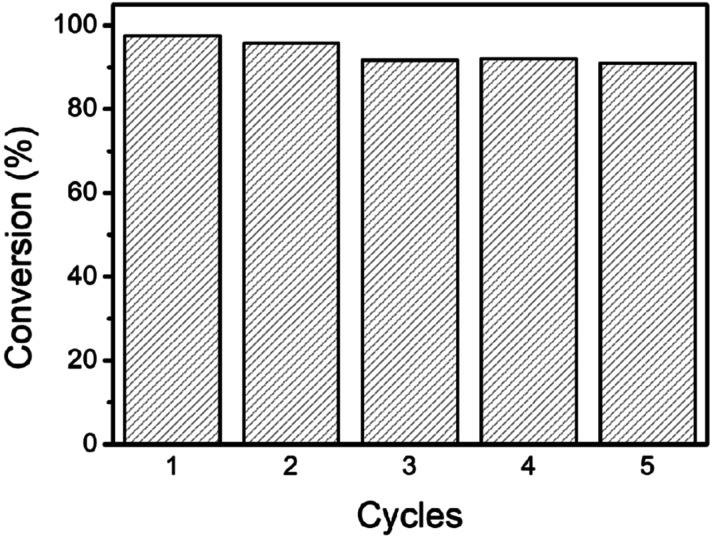
The reusability of Ag/ZnO/CaCO_3_ in the reduction of 4-NP with NaBH_4_.

Due to its poor solubility and high stability, 4-NP is considered to be one of the most troublesome water pollutants, which is often degraded by NaBH_4_ with the addition of catalysis.^37–39^ Therefore, the reduction of 4-NP was selected as the model reaction to evaluate the performance of Ag/ZnO/CaCO_3_ for the wastewater treatment. As shown in Fig. 6a, the absorption peak at 400 nm ascribed to 4-NP rapidly decreased with time and completely disappeared after 8 minutes over Ag/ZnO/CaCO_3_. In addition, two new peaks at 300 nm and 230 nm simultaneously appeared, which are assigned to the characteristic peaks of 4-AP. These observations suggested that 4-NP can be reduced to 4-AP over Ag/ZnO/CaCO_3_ in an efficient manner. As compared to the binary Ag/CaCO_3_ and ZnO/CaCO_3_, Ag/ZnO/CaCO_3_ exhibited a better catalytic degradation performance by showing the highest degradation rate at 97.55% (Fig. 6b). The time-dependent reduction of 4-NP suggested that the reduction of 4-NP is extremely sensitive over the as-obtained Ag/ZnO/CaCO_3_. The pseudo-first-order kinetic model (d_*C*_/d_*t*_ = *k*) was used to calculate the kinetic constant (*k*). The plot of ln(*C*/*C*_0_) and the reaction time showed that the reduction of 4-NP over the as-obtained Ag/ZnO/CaCO_3_ followed a pseudo-first-order kinetic model, and the *k* value of this system was 0.4667 min^−1^. In addition, the as-obtained Ag/ZnO/CaCO_3_ showed high stability in the reaction. Five cycling tests revealed no obvious decrease of the activity (Fig. 7).

The mechanism for the 4-NP reduction is proposed based on the already established fact.^40^ First, it is noted that the 4-NP solution changes from a light yellow color to bright yellow green after adding NaBH_4_, which is caused by the formation of 4-nitrophenolate ions. The absorption peak of the 4-NP solution also shifts from 317 nm to 400 nm.^13,37^ The 4-nitrophenolate ion and BH^4−^ are adsorbed onto the surface of Ag/ZnO *via* electrostatic interactions and/or van der Waals' forces to free up the active site.^41^ During the process, the BH^4−^ radical is responsible for transferring hydrogen to Ag NPs and ZnO NPs, and hence resulting in a reaction that forms two intermediates: an unstable 4-nitrosophenol and a stable 4-hydroxylaminophenol.^42^ The as-formed 4-hydroxylaminophenol is further activated to produce the final product 4-AP, which is visualized by the color changes from a yellow-green color to transparent. The amounts of active sites are normally considered the rate-determining step. This clearly indicates a strong synergistic effect between the Ag NPs and ZnO NPs, which facilitates the electron transfer relevant for 4-NP reduction.^43^

### 3.6 Antibacterial activity

**Fig. 8:**
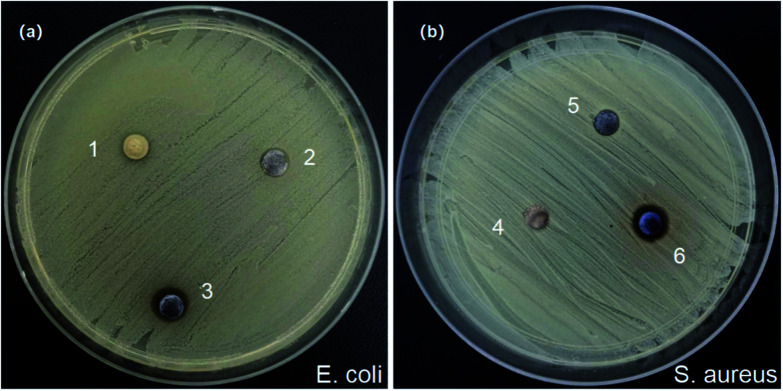
Photographs of the zone of inhibition against (a) *E. coli* and (b) *S. aureus* by Ag/CaCO_3_, ZnO/CaCO_3_, and Ag/ZnO/CaCO_3_ nanomaterials. (1–3 and 4–6 represent the addition of ZnO/CaCO_3_, Ag/CaCO_3_ and Ag/ZnO/CaCO_3_ nanocomposites after 24 hours incubation.)

It has been found that ZnO NPs showed marked antibacterial activity through the production of reactive oxygen species (ROS) and the release of Zn^2+^.^44,45^ Therefore, the antibacterial activity of Ag/ZnO/CaCO_3_ was also studied. In this part, the *E. coli* and *S. aureus* bacteria were used as experimental strains to evaluate the antibacterial activity of Ag/ZnO/CaCO_3_. Compared with binary Ag/CaCO_3_ and ZnO/CaCO_3_, Ag/ZnO/CaCO_3_ exhibited an effective antibacterial activity for both *E. coli* and *S. aureus* by showing larger inhibition zones of *E. coli* and *S. aureus* (Fig. 8a and b). In other words, the synergistic effect among the Ag, ZnO and CaCO_3_ played an important role in improving its antibacterial properties.

**Fig. 9:**
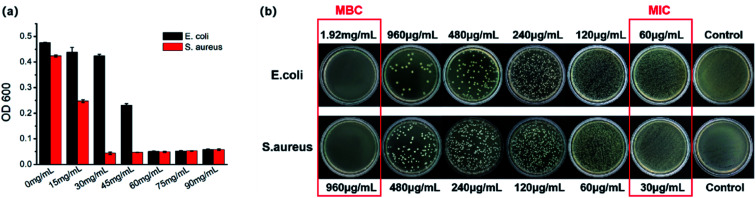
Comparative plots show changes with Ag/ZnO/CaCO_3_ nanocomposites at different concentrations against *E. coli* and *S. aureus*.

The optical density was measured at 600 nm to analyze the antimicrobial efficiency of different concentrations of the Ag/ZnO/CaCO_3_ nanocomposites. As shown in Fig. 9a, the Ag/ZnO/CaCO_3_ nanocomposites at concentrations of 60 μg mL^−1^ and 30 μg mL^−1^ can completely inhibit the growth of *E. coli* and *S. aureus*, respectively. In addition, the MBC test concentration was selected on the foundation of the results of the MIC experiment. As the results show in Fig. 9b, no *E. coli* colony was formed on the culture plate at a concentration of 1.92 mg mL^−1^, and the colony-forming units of the *S. aureus* bacteria were reduced to zero at a concentration of 960 μg mL^−1^, implying that both *E. coli* and *S. aureus* can be completely killed over Ag/ZnO/CaCO_3_. This may be related to the special cell membrane structure of *E. coli* that resists the entry of antimicrobial agents.^46,47^

**Fig. 10:**
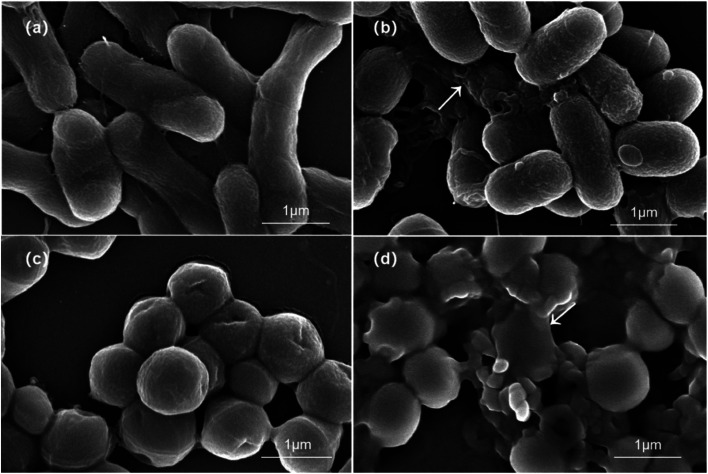
SEM images of the status of *E. coli* and *S. aureus* before (a and c) and after treatment (b and d) with the Ag/ZnO/CaCO_3_ nanocomposites.

The SEM images of *E. coli* further revealed that untreated *E. coli* bacteria exhibited typical rod morphology with the intact surfaces and peripheral flagella. When co-cultured with the samples, the *E. coli* bacteria became short and rough, leading to the shrinkage of cells. Similarly, the initial *S. aureus* were round-shaped with smooth and intact cell walls. However, they showed membrane damage and distortion after treatment, resulting in the leakage of intracellular components and finally cell lysis. The damaged morphology was attributed to the internalization of the Ag/ZnO/CaCO_3_ nanocomposites. Generally, the release of metal ions and the production of ROS are two main factors affecting the structures of cells, leading to irreversible DNA damage and subsequent bacterial cell death (Fig. 10).

**Fig. 11:**
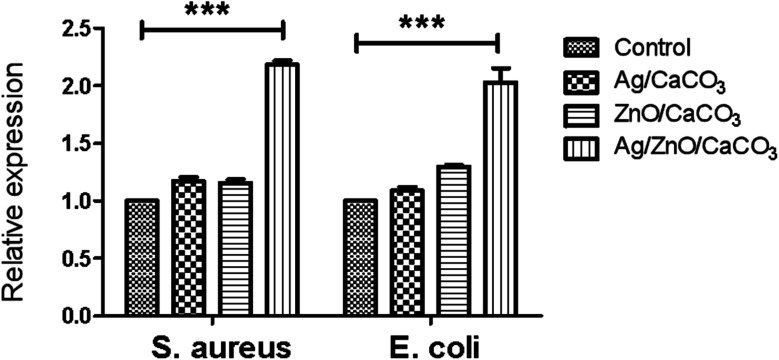
Relative expression of ROS in *E. coli* and *S. aureus* with different materials (Ag/CaCO_3_, ZnO/CaCO_3_ and Ag/ZnO/CaCO_3_) and without treatment, using fluorescence spectroscopy. (***: *P* < 0.01).

In order to investigate the effect of ROS on the bactericidal mechanism, the DCDFH-DA fluorescent probe was used to detect the production of ROS in bacterial cells treated with different materials.^48,49^ Data were normalized by relative expression and statistical analysis by the Student's *t* test. As compared to the binary Ag/CaCO_3_ and ZnO/CaCO_3_, Ag/ZnO/CaCO_3_ produced an approximate 2-fold increase of ROS. All of these studies revealed that the Ag/ZnO/CaCO_3_ nanocomposites were capable of producing ROS, and resulted in irreversible DNA damage and bacteria death (Fig. 11).^50^

## 4. Conclusions

In summary, Ag/ZnO coated on oyster shell-derived CaCO_3_*via Cacumen platycladi* extract (Ag/ZnO/CaCO_3_) exhibited excellent performance for the reduction of 4-nitrophenol (4-NP) with a reduction rate at 97.6% within 8 minutes. It also demonstrated superior antibacterial activity in inhibiting the growth of *E. coli* and *S. aureus* with the minimum inhibition concentrations (MIC) of 60 μg mL^−1^ and 30 μg mL^−1^, respectively, which is attributed to the synergistic effect of Ag, ZnO and the oyster shell-derived CaCO_3_. This study not only provides a strategy for developing highly efficient catalysts for the reduction of 4-NP and an antibacterial agent based on oyster shell-derived CaCO_3_, but also highlights the great potential of oyster shell-derived CaCO_3_ for wastewater treatment.

## Conflicts of interest

There are no conflicts to declare.

## Supplementary Material

RA-009-C9RA08960H-s001
