# Supplementary material for: Dual functional oyster shell-derived Ag/ZnO/CaCO3 nanocomposites with enhanced catalytic and antibacterial activities for water purification
Source: RSC Adv. 2019 Dec 13;9(70):41336–44. doi: 10.1039/c9ra08960h (PMC9076377; doi:10.1039/c9ra08960h)
Supplement: RA-009-C9RA08960H-s001 [file RA-009-C9RA08960H-s001.pdf]

Electronic Supplementary Material (ESI) for RSC Advances.
This journal is © The Royal Society of Chemistry 2019

Supporting Information

Dual Functional Oyster Shell-derived $Ag/ZnO/CaCO_3$ Nanocomposites with Enhanced Catalytic and Antibacterial Activities for Water Purification

Lin Chang [a,b§], Yan Feng [a,b§], Xiaoyu Huang [a,b], Youguang Lu [a,b*], Da-Peng Yang [c*]

[a] Department of Preventive Dentistry, School and Hospital of Stomatology, Fujian Medical University, Fuzhou 350002, Fujian Province, PR China.

[b] Key Laboratory of Stomatology & Fujian Provincial Engineering Research Center of Oral Biomaterial, School and Hospital of Stomatology, Fujian Medical University, Fuzhou 350004, Fujian Province, PR China.

[c] College of Chemical Engineering and Materials Science, Quanzhou Normal University, Quanzhou 362000, Fujian Province, PR China.

[§] Equal contribution

* Corresponding Authors:

Dr. Youguang Lu

Phone: +86-591-83736429; Fax: +86-591-83720599

Email address: fjlyg63@163.com

Dr. Da-Peng Yang

Tel.: +86-595-22199816; fax: +86-595-22199816

E-mail address: yangdp@qztc.edu.cn

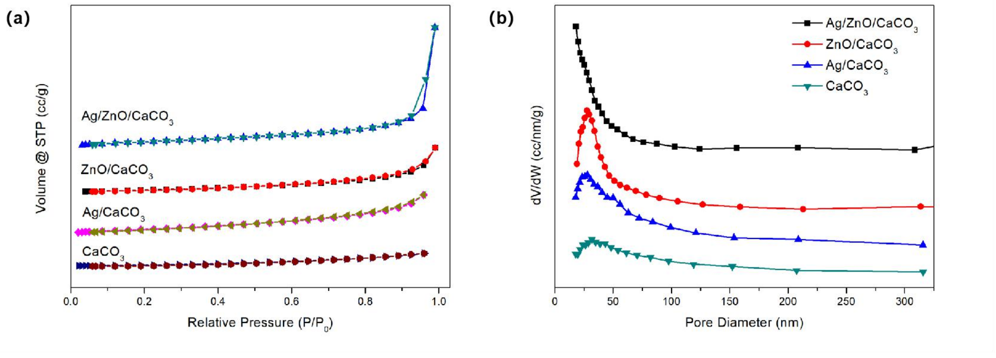

Fig. S1. N2 adsorption-desorption isotherms (a) and pore size distribution (b) of $CaCO_3$, $Ag/CaCO_3$, $ZnO/CaCO_3$,and $Ag/ZnO/CaCO_3$ nanocomposites.

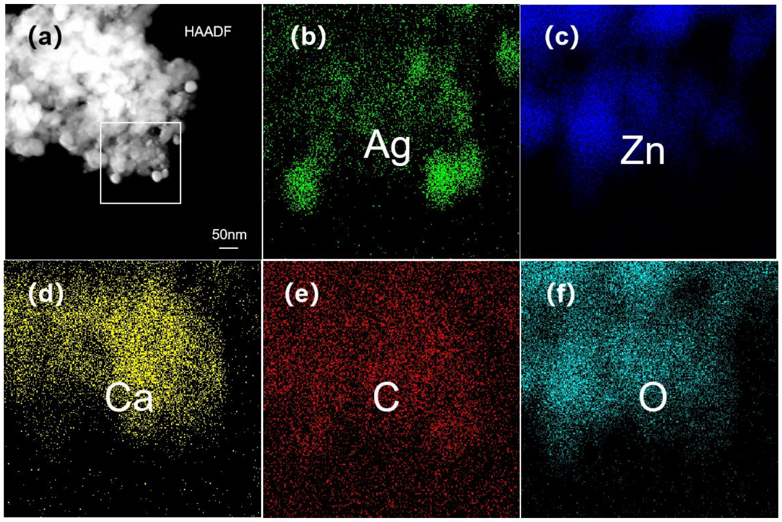

Fig. S2. TEM images of $Ag/ZnO/CaCO_3$ and corresponding elemental mapping images showing Ag, Zn, Ca, C and O in the selected area.
